# Risk Factors for Enterotoxigenic *Bacteroides fragilis* Infection and Association with Environmental Enteric Dysfunction and Linear Growth in Children: Results from the MAL-ED Study

**DOI:** 10.4269/ajtmh.21-0780

**Published:** 2022-01-31

**Authors:** Parag Palit, Rina Das, Md Ahshanul Haque, Sharika Nuzhat, Shaila Sharmeen Khan, Towfida Jahan Siddiqua, Mustafa Mahfuz, Abu Syed Golam Faruque, Tahmeed Ahmed

**Affiliations:** ^1^ Nutrition and Clinical Services Division, International Center for Diarrheal Disease Research, Bangladesh, Dhaka, Bangladesh;; ^2^ Emerging Infections and Parasitology Laboratory, International Center for Diarrheal Disease Research, Bangladesh, Dhaka, Bangladesh;; ^3^ Faculty of Medicine and Life Sciences, University of Tampere, Tampere, Finland;; ^4^ James P. Grant School of Public Health, BRAC University, Dhaka, Bangladesh

## Abstract

Despite reports of enterotoxigenic *Bacteroides fragilis* (ETBF) isolation from asymptomatic children, no reports exist regarding the possible association of ETBF with long-term complications such as development of environmental enteric dysfunction (EED) and subsequent linear growth faltering in childhood. We aimed to establish a potential association between the burden of asymptomatic ETBF infection and EED and linear growth at 24 months of age using the data collected from 1,715 children enrolled in the multi-country birth cohort study, known as the Etiology, Risk Factors, and Interactions of Enteric Infections and Malnutrition and the Consequences for Child Health study. Using Poisson regression models, we evaluated the site-specific incidence rate and, subsequently, identified the risk factors and assessed the association between the burden of ETBF infection and EED score and linear growth at 24 months of age. The overall incidence rate of ETBF infections per 100 child-months across all study sites was 10.6%, with the highest and lowest incidence of ETBF infections being reported in Tanzania (19.6%) and Peru (3.6%), respectively. Female gender, longer duration of breastfeeding, and improved water access, sanitation, and hygiene practices, such as improved drinking water source, improved sanitation, and improved floor material in households, along with enhanced maternal education and less crowding in the households were found to be protective against incidences of ETBF infection. The burden of ETBF infections was found to have significant associations with EED and linear growth faltering at 24 months of age across all the study sites. Our findings warrant regular clinical monitoring to reduce the burden of ETBF infections and diminish the burden of enteropathy and linear growth faltering in childhood.

## INTRODUCTION

Childhood malnutrition accounts for approximately 45% of the cases of “under-five” mortality and has been reported to be associated strongly with a number of acute illnesses and severe damage to several physiological activities, including restricted mental growth and development in later life, cardiovascular and chronic respiratory illnesses, and compromised host immunity.^1^^,^^2^ Stunting or linear growth faltering (length-for-age z-score [LAZ] < 2 points of the WHO growth standard) is the most prevalent form of childhood malnutrition and affects an estimated 149 million children worldwide.^3^^,^^4^ The first 2 years of life are a critical window for long-term growth and development, and stunting has been reported to exhibit extremely serious and adverse consequences in children younger 2 years of age.^5^ Repeated exposure to fecal enteropathogens in early life and subsequent development of environmental enteric dysfunction (EED) are considered to be the primary factors attributing to linear growth failure in the early years of life.^6^

EED refers to functional changes in the gut, including influx of inflammatory cells to the intestine and consequent local and systemic inflammation resulting from damage to intestinal epithelial cells, increased permeability, and microbial translocation into the lamina propria as a result of a number of environmental determinants, which are often reversible.^7^ Chronic exposure to enteropathogens leading to persistent immune activation, increased intestinal permeability, and enteric inflammation are hallmarks of EED.^8^^,^^9^ Small intestine biopsy is considered to be the gold standard for the diagnosis of EED, but it is not feasible to perform in children because of the invasiveness protocols involved with the procedure.^10^ Consequently, several fecal biomarkers—namely, myeloperoxidase (MPO), neopterin (NEO), and α-1-antitrypsin (AAT)—have been reported to be associated with EED and have been proposed as noninvasive alternatives for the assessment of EED.^11^^,^^12^ Among these biomarkers in stool, MPO and NEO are markers of intestinal inflammation, whereas AAT indicates loss of serum proteins into the intestinal lumen as a result of altered intestinal permeability.^13^

Enterotoxigenic *Bacteroides fragilis* (ETBF) is an enteropathogen that causes diarrhea in both children and adults, and is one of the leading anaerobic isolates in clinical specimens.^14^ The prevalence of the association of ETBF with diarrhea has been reported to vary across geographic locations, ranging from 3.5% in Bangladesh to 28% in Italy.^15^ Sack et al.^16^ reported ETBF to be an important etiological agent in acute diarrhea in children older than 1 year in an age-structured case–control study. ETBF is known to produce a 20-kDa zinc-dependent, non-lethal, heat-labile metalloprotease known as “fragilysin,” which acts by cleaving the E-cadherin protein of zonula adherens and tight junctions, potentially leading to altered intestinal permeability.^17^^,^^18^ This toxin has been reported to result in swelling and rounding of the cultured enteric cell lines.^19^^,^^20^ Subsequent findings from a study conducted among children and adults with acute diarrheal illnesses in Dhaka, Bangladesh, indicate that ETBF induces intestinal inflammation.^21^ In one report, two to four sequential episodes of ETBF-associated diarrhea were reported for children in Bangladesh, suggesting that acquired immunity to ETBF is incomplete.^22^

Similar to the case for other enteric pathogens, asymptomatic ETBF colonization is common and the prevalence ranges from 4% to 20% for asymptomatic ETBF infection.^15^^,^^23^ Findings from an endoscopy-based study reported that ETBF was recovered from 35% of stool samples of control patients without diarrhea.^24^ However, the relationship between the burden of ETBF infection on the potential development of enteric inflammation and subsequent linear growth failure has not been reported. Therefore, we conducted this study using data from a multi-country birth cohort study to estimate incidence rates of ETBF infections across different geographic locations, and to establish a possible relationship between EED and linear growth among children at 24 months of age.

## MATERIALS AND METHODS

### Study design and ethical statement.

The study design of the multi-country birth cohort Etiology, Risk Factors, and Interactions of Enteric Infections and Malnutrition and the Consequences for Child Health (MAL-ED) study, conducted across eight sites (Bangladesh, Brazil, India, Nepal, Peru, Pakistan, South Africa, and Tanzania) in South America, sub-Saharan Africa, and Asia have been described previously.^25^ In short, 1,715 children were enrolled from November 2009 to February 2012 from the community within 17 days of birth at all eight sites and were monitored up to 24 months of age. The study was approved by the respective institutional review boards at each of the eight study sites.^25^ Written, informed voluntary consent was obtained from the parents or legal guardians of every child.

### Collection of anthropometric, sociodemographic, and morbidity data.

Anthropometric indices of LAZ, weight-for-age z-score, and weight-for-length z-score were calculated according to 2006 WHO standards for children^26^ using data of monthly anthropometric measurements done up to the age of 24 months, using standard scales (seca GmbH & Co. KG, Hamburg, Germany). Sociodemographic data on the child’s birth, including birthweight, anthropometric indices at birth, presence of siblings, and other maternal characteristics, were collected at enrollment.^25^ A detailed account of any morbidity and child feeding practices was obtained during household visits, which were conducted twice weekly.^27^

Socioeconomic data were collected every 6 months, starting from 6 months of age of the participant. The water, sanitation, hygiene, asset, maternal education, and income index (WAMI), which ranges from 0 to 1 point, is an index of socioeconomic status of the households^28^ and was calculated subsequently. Greater socioeconomic status was indicated by a higher WAMI.^29^ WHO guidelines were followed to define improved water and sanitation,^30^ whereas treatment of drinking water was defined as filtering, boiling, or adding bleach.^31^

### Collection of stool and blood samples.

Across all the study sites, non-diarrheal stool samples were collected each month (at least 3 days before or after a diarrhea episode) after enrollment and up to the age of 24 months. No diarrheal samples were analyzed in this study. Venous blood samples were collected at 7, 15, and 24 months of age.^32^ After collecting these biological samples, they were processed in the laboratories across all the sites according to the same standardized procedures and were stored in –80 °C freezers before subsequent laboratory analysis.^33^

Plasma zinc, a proxy marker that is also recommended for assessment of zinc status in children,^34^ was assessed at 7, 15, and 24 months using lame atomic absorption spectrophotometry (Shimadzu AA-6501S, Kyoto, Japan).^35^ α-1-Acid glycoprotein (AGP) in plasma, a biomarker for systemic inflammation,^36^ was assessed at 7, 15, and 24 months using an immunoturbidimetric assay with commercial kits from Roche Diagnostics on a Roche automated clinical chemistry analyzer (Hitachi-902, Boehringer Mannheim, Germany).

### Evaluation of biomarkers of enteric inflammation and calculation of EED score.

Enteric inflammation was ascertained by measuring the levels of the inflammatory biomarkers MPO (Alpco, Salem, NH), NEO (GenWay Biotech, San Diego, CA), and AAT (Biovendor, Chandler, NC) in the stool samples collected from the study participants at 3, 6, 9, 15, and 24 months by quantitative ELISA using manufacturer guidelines.^25^ At each time point, the EED score, ranging from 0 to 10 points, was calculated from the three fecal markers, as described in previous studies.^37^^,^^38^ Categories were assigned the values 0 point (low), 1 point (medium), or 2 points (high). MPO, NEO, and AAT values were log‐transformed prior to subsequent analysis. The formula used for the calculation of the EED score is as follows:^37^

EED score=2×AAT category+2×MPO category+1×NEO category.

### Assessment of enteropathogens by TaqMan array cards.

TaqMan array cards, customized multiplex quantitative polymerase chain reaction involving compartmentalized primer-probe assays, were used to detect a possible 29 pathogens from each of the samples, using protocols described elsewhere.^39^^,^^40^ The quantification cycle value of 35 was set as a threshold for analysis, as mentioned elsewhere.^39^ In our study, we investigated the occurrence of the *bft* gene of ETBF, and positive cases were analyzed further for co-infection for the presence of distinct other co-pathogens—namely, *Campylobacter* sp., enterotoxigenic *Escherichia coli* (ETEC), enteroaggregative *E. coli* (EAEC), typical enteropathogenic *E. coli* (tEPEC), *Shigella*/enteroinvasive *E. coli*, *Cryptosporidium* spp. and *Giardia* spp., as described previously.^41^

### Statistical analysis.

All statistical analyses were performed using STATA V13 (Stata Corp. LLC, College Station, TX). Data were summarized as either the mean and sd or as the median with the interquartile range, depending on the distribution of the data. Poisson regression was used to calculate the incidence rates for ETBF infection, whereby the number of ETBF infections was considered the outcome variable and the log of several follow-ups was considered the offset variable.

Determinants for the monthly detection of ETBF in the stool samples were assessed using Poisson regression models, whereby the variables that were included in the final model, adjusting for all study sites and using stepwise forward selection, were gender of the participant, duration of exclusive breast feeding in months, LAZ at enrollment, maternal height, maternal age, maternal education, improved floor, access to an improved drinking water source, routine treatment of drinking water, lack of improved sanitation, more than two people living per room, mother having more than three living children, and household ownership of chicken or cattle.

The burden of ETBF infections was defined as the number of ETBF infections over the total number of follow-ups conducted throughout the study period. Associations between the burden of ETBF infection and linear growth and EED score at 24 months of age was determined using multivariate linear regression after adjusting for potential covariates—namely, gender of the participant, exclusive breast feeding, LAZ at enrollment, maternal age, maternal height, WAMI, more than two people living per room, mother having more than three living children, household ownership of chicken or cattle, plasma AGP level at 24 months, plasma zinc level at 24 months, and burden of other malnutrition associated co-pathogens during the study period (*Campylobacter*, ETEC, EAEC, tEPEC, *Shigella* sp., *Cryptosporidium*, and *Giardia*) for the overall estimate. For the growth analysis, we excluded the data collected from the participants enrolled at the Pakistan site because of bias noted at this site during the study period. Because the aim of our study was to analyze the possible association between burden of ETBF infection with enteric inflammation and linear growth at 24 months, we considered the plasma AGP and zinc levels at 24 months only.

The selection of the variables for the multivariate analysis was based on prior literature review,^42^ after which we prepared a conceptual framework for factors influencing enteric inflammation and subsequent linear growth from birth up until 2 years of life. Multicollinearity between the variables adjusted in the variance inflation factor was calculated, and no variable producing a variance inflation factor value of more than the threshold of five was found in the final model. The strength of association was evaluated by estimating the β-coefficient and its corresponding 95% CI. *P* < 0.05 was considered statistically significant during the analysis.

## RESULTS

A total of 1,715 participants were enrolled across the eight different study sites, and a total of 34,622 monthly non-diarrheal stool samples were collected from the study participants throughout the entire study period, starting from their enrollment after birth. Table 1 shows the general characteristics of the study participants enrolled at each of the eight study sites.

**Table 1:** General characteristics of the study participants enrolled at all the eight study sites

Characteristics	Bangladesh (*n* = 210)	Brazil (*n* = 165)	India (*n* = 227)	Nepal (*n* = 227)	Peru (*n* = 194)	Pakistan (*n* = 246)	South Africa (*n* = 237)	Tanzania (*n* = 209)
Male gender (n, %)	108 (51.4)	89 (53.94)	105 (46.3)	122 (53.7)	105 (54.1)	120 (48.8)	120 (50.6)	105 (50.2)
Birthweight, kg	2.8 ± 0.4	3.4 ± 0.5	2.9 ± 0.4	3.0 ± 0.4	3.1 ± 0.4	2.7 ± 0.4	3.2 ± 0.5	3.2 ± 0.5
LAZ at enrollment	–1.0 ± 1.0	–0.8 ± 1.1	–1.0 ± 1.1	–0.7 ± 1.0	–0.9 ± 1.0	–1.3 ± 1.1	–0.7 ± 1.0	–1.0 ± 1.1
LAZ at 24 mo	–2.0 ± 0.9	0.0 ± 1.1	–1.9 ± 1.0	–1.3 ± 0.9	–1.9 ± 0.9	–	–1.7 ± 1.1	–2.7 ± 1.0
WAZ at enrollment	–1.3 ± 0.9	–0.2 ± 1.0	–1.3 ± 1.0	–0.9 ± 1.0	–0.6 ± 0.9	–1.4 ± 1.0	–0.4 ± 1.0	–0.1 ± 1.1
WAZ at 24 mo	–0.8 ± 0.9	0.5 ± 1.4	–0.9 ± 0.9	–0.3 ± 0.9	0.3 ± 0.9	–	0.5 ± 1.0	0.1 ± 1.0
Days of exclusive breastfeeding	143.2 ± 42.7	93.7 ± 57.8	105.4 ± 42.9	92.5 ± 54.5	89.5 ± 61.3	19.9 ± 22.7	38.6 ± 26.3	62.2 ± 35
Maternal age during enrollment, y	25 ± 5	25.4 ± 5.6	23.9 ± 4.2	26.6 ± 3.7	24.8 ± 6.3	28.1 ± 5.9	27 ± 7.2	29.1 ± 6.5
Maternal BMI during enrollment	22.3 ± 3.4	25.7 ± 4.4	22.0 ± 4.0	25.1 ± 3.2	24.9 ± 3.7	21.5 ± 3.8	27 ± 5.5	22.9 ± 3.2
Maternal education > 6 y of schooling*, n* (%)	77 (36.7)	413 (86.7)	147 (64.8)	168 (74)	150 (77.3)	44 (17.9)	233 (97.9)	134 (64.1)
Presence of more than 3 living children at household during enrollment*, n* (%)	160 (76.2)	113 (68.5)	157 (69.8)	199 (87.7)	111 (57.2)	105 (42.7)	141 (59.5)	58 (27.8)
More than 2 people living per room, *n* (%)	202 (96.2)	24 (14.5)	181 (79.7)	101 (44.5)	72 (37.1)	219 (89.1)	36 (15.2)	114 (54.5)
Improved drinking water source, *n* (%)	210 (100)	165 (100)	227 (100)	227 (100)	184 (94.9)	246 (100)	196 (82.7)	89 (42.6)
Improved floor, *n* (%)	204 (97.1)	165 (100)	222 (97.8)	109 (48)	69 (35.6)	81 (32.9)	231 (97.5)	13 (6.2)
Average plasma zinc level, mmol/L; median (IQR)	11.3 (10.6– 12.1)	14 (13– 14.9)	9.1 (8.6– 9.6)	11.2 (10.4–12.2)	14.8 (13.1–17.9)	8.9 (7.7–10)	22.9 (14.3–32.9)	11.1 (9.9–12.3)
Average plasma AGP, mg/dL; median, (IQR)	84.3 (71.5– 105.3)	95.7 (81– 117)	97 (83–110)	117.7 (102.7– 139)	115 (98– 130.3)	93 (77.5– 111.8)	126 (107.3–153.7)	114.3 (97.7–138.7)

AGP = α-1-acid glycoprotein; BMI = body mass index; IQR = interquartile range; LAZ = length-for-age z-score, WAZ = weight-for-age z-score, WHZ = weight-for-height z-score. Data are presented as mean ± sd unless otherwise mentioned.

The proportion of male participants was the highest in Peru and the lowest in India, whereas birthweight was the highest in Brazil and the lowest in Pakistan. The longest duration of exclusive breastfeeding was found in Bangladesh; the shortest duration was found in Pakistan. Maternal education (as defined by more than 6 years of schooling) was the highest in South Africa and lowest in Pakistan. Access to improved drinking water in the households of all enrolled study participants was reported in Bangladesh, Brazil, India, Nepal, and Pakistan. The highest average plasma zinc and plasma AGP levels throughout the study period was found in South Africa.

**Figure 1.**
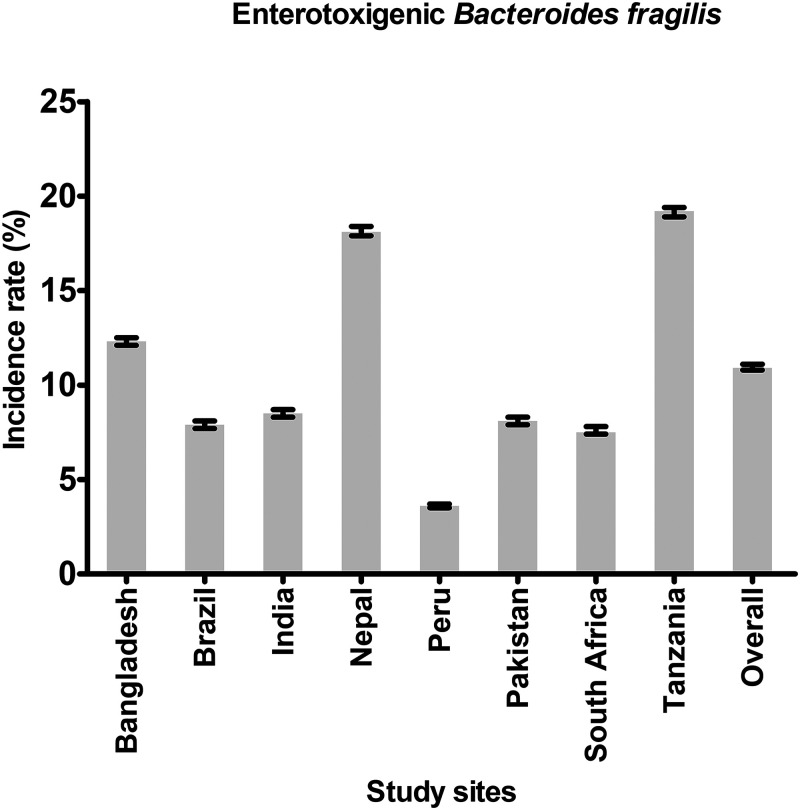
Incidence rates of enterotoxigenic *Bacteroides fragilis* per 100 child-months among the participants enrolled at each of the eight study sites. Error bars = 95% CI.

The incidence rates of ETBF infection per 100 child-months across each of the eight study sites over the 24-month study period is shown in Figure 1. The overall incidence rate of ETBF infection across all eight study sites was 10.9%. The highest incidence rate of ETBF infection per 100 child-months was found in Tanzania (19.2%); the lowest incidence rate was found in Peru (3.6%).

### Sociodemographic factors associated with ETBF infection.

Table 2 shows the relationship between different sociodemographic factors and ETBF infection across the eight different study sites. Female gender was found to have a significantly lower association with the incidence rate of ETBF infection per 100 child-months. Duration of exclusive breastfeeding, maternal height, maternal age, and routine treatment of drinking water did not have any association with the incidence rate of ETBF infection. However, increased maternal education, access to improved drinking water, and improved floor material in the households were found to be associated with a significantly lower incidence rate of ETBF infection per 100 child-months. Consequently, other sociodemographic factors such as lack of access to improved sanitation, more than two people living per room, mother having more than three living children, and household ownership of chicken or cattle were found to be significantly associated with an increased incidence rate of ETBF infection per 100 child-months.

**Table 2:** Sociodemographic factors associated enterotoxigenic *Bacteroides fragilis* infection in each of the eight study sites

Factors	Incidence rate ratio of *B. fragilis* detection per 100 child-months (95% CI)	*P* value
Female gender	0.96 (0.94–0.97)	< 0.001
Duration of exclusive breastfeeding, mo	0.98 (0.96–0.99)	< 0.001
LAZ at enrollment	0.98 (0.97–0.99)	< 0.001
Maternal height, cm	1.0 (0.99–1.0)	0.352
Maternal age, y	1.0 (0.99–1.0)	0.004
Maternal education > 6 y	0.89 (0.88–0.91)	< 0.001
Improved drinking water source	0.92 (0.90–0.95)	< 0.001
Routine treatment of drinking water	1.0 (0.98–1.02)	0.732
Lack of improved sanitation	1.21 (1.18–1.25)	< 0.001
Improved floor material in households	0.93 (0.90–0.95)	< 0.001
More than 2 people living in a single room	1.04 (1.02–1.05)	< 0.001
Mother has more than 3 living children	1.10 (1.07–1.11)	< 0.001
Household ownership of chicken or cattle	1.15 (1.13–1.17)	< 0.001

LAZ = length-for-age z-score. A Poisson regression model was used. The dependent variable was the number of infections during follow-up (1–24 months) and the offset variable was the log of the total number of follow-ups. All analyses were adjusted for different study sites and all variables included in the multivariable model.

### Association between the burden of ETBF infection and enteric inflammation and linear growth at 24 months of age.

Table 3 shows the results of a separate analysis involving the association between the burden of ETBF infection with enteric inflammation and linear growth at 24 months of age. After adjusting for variables such as gender of the participant, exclusive breastfeeding, LAZ at enrollment, maternal age, maternal height, WAMI, more than two people living per room, mother having more than three living children, household ownership of chicken or cattle, plasma AGP level at 24 months, plasma zinc level at 24 months, and presence of other malnutrition associated co-pathogens (*Campylobacter* sp., ETEC, EAEC, tEPEC, *Shigella* sp., *Cryptosporidium* sp. and *Giardia* sp.), the burden of ETBF infection over the study period across all eight study sites was found to have a positive association with enteric inflammation at 24 months of age, as expressed by the EED score. Consequently, linear growth at 24 months as expressed by LAZ was found to be associated negatively with the burden ETBF infection over the study period across all eight study sites. Our findings indicate that with a 1-U increase in the burden of ETBF infection, the EED score at 24 months would be likely to increase by 0.256 U, and LAZ at 24 months would be likely to decrease by 0.155 U.

**Table 3:** Association between burden of enterotoxigenic *Bacteroides fragilis* infection on enteric inflammation and linear growth at 24 months of age

Outcome variable	EED score at 24 months		LAZ at 24 months	
	Adjusted β-coefficient, 95% CI	*P* value	Adjusted β-coefficient (95% CI)	*P* value
Burden of ETBF infection	0.256	0.024	–0.155 (–0.22 to –0.083)	< 0.001
Male gender	–0.275	< 0.001	–00.243	< 0.001
Exclusive breastfeeding	0.026	0.024	–00.051	< 0.001
LAZ at enrollment	–0.197	< 0.001	0.298	< 0.001
Maternal age	0.001	0.438	0.008	0.512
Maternal body mass index	0.170	< 0.001	0.320	< 0.001
WAMI index	–0.458	0.007	0.946	< 0.001
Mother having more than 3 living children	0.023	0.647	–00.156	< 0.001
More than 2 people living in a room	0.233	< 0.001	–0.120	< 0.001
Household ownership of chicken or cattle	0.147	0.011	–0.078	< 0.001
AGP level at 24 mo	0.065	< 0.001	–0.111	< 0.001
Plasma zinc at 24 mo	–0.129	< 0.001	0.083	< 0.001
Burden of *Campylobacter* infection	0.712	< 0.001	–0.198	< 0.001
Burden of ETEC infection	0.741	< 0.001	–0.244	< 0.001
Burden of EAEC infection	1.108	< 0.001	–0.300	< 0.001
Burden of typical EPEC infection	0.737	0.002	–0.360	< 0.001
Burden of *Shigella* infection	0.889	< 0.001	–0.419	< 0.001
Burden of *Cryptosporidium* infection	0.865	0.005	–0.320	< 0.001
Burden of *Giardia* infection	0.387	< 0.001	–0.367	< 0.001

AGP = α-1-acid glycoprotein; EAEC = enteroaggregative *Escherichia coli*; EED = environmental enteric dysfunction; EPEC = enteropathogenic *Escherichia coli*; ETBF = enterotoxigenic *Bacteroides fragilis*; ETEC = enterotoxigenic *Escherichia coli*; WAMI = water/sanitation, assets, maternal education, and income; LAZ = length-for-age z-score. The final regression model was adjusted for gender of the participant, exclusive breastfeeding, LAZ at enrollment, maternal age, maternal body mass index, WAMI index, more than two people living per room, mother having more than three living children, household ownership of chicken or cattle, plasma AGP level at 24 months, plasma zinc level at 24 months, and presence of co-pathogens (*Campylobacter* sp., ETEC, EAEC, typical EPEC, *Shigella* sp., *Cryptosporidium* sp., and *Giardia* sp.) for the overall estimate.

## DISCUSSION

Findings from previous studies conducted in the settings of the MAL-ED study have reported that linear growth faltering at 24 months of life was attributed to asymptomatic infections by *Shigella* sp., EAEC, *Campylobacter* sp.,* Giardia* sp.,* Cryptosporidium* sp., and tEPEC.^40^ However, to the best of our knowledge, this is the first study that has aimed to estimate incidence rates of ETBF infections across different geographic locations and establish a possible association between EED and linear growth among children at 24 months of age. Diagnosis of ETBF infection from stool is difficult, with anaerobic stool culture for *B. fragilis* being delayed by the processing of stool samples, along with the general difficulty of anaerobic microbiology and fecal heterogeneity of *B. fragilis* strains, whereby both non-enterotoxigenic *B. fragilis* and ETBF may be detected in the stool samples.^43^^–^^45^ Thus, detection of the *bft* gene by polymerase chain reaction is recommended for accuracy in the diagnosis of ETBF infection.^15^

Reports from studies conducted in India, Bangladesh, and Brazil found that the prevalence of ETBF among acute diarrheal cases was between 2% and 4%.^46^^–^^48^ From our analysis, we report a 10.9% overall incidence rate of *B. fragilis* infection per 100 child-months across all eight study sites, with the highest incidence rate per 100 child-months in Tanzania (19.2%) and the lowest incidence rate per 100 child-months in Peru (3.6%). Findings from a case–control study conducted among children in India show that the prevalence of ETBF in asymptomatic cases was 7.2%.^49^ Reports from another study conducted in a rural area of Bangladesh show that approximately 16% of all children younger than 24 months of age were infected with ETBF at some time after birth.^22^ However, all these aforementioned studies were not longitudinal in design and did not involve monthly follow-ups for the assessment of the burden of enteropathogens and did not involve the use of such a sophisticated molecular diagnostic technique such as the TaqMan array cards, as did our study.

Reports involving the risk factors for ETBF are very limited. One study carried out in rural Bangladesh indicated that household ownership of cattle or poultry was a determinant of ETBF infection among children.^22^ Previous reports indicate that different domestic animals, including lambs and calves, can have ETBF-associated diarrhea.^50^^,^^51^ The results from our study show that the household ownership of chicken or cattle was significantly associated with a 15% higher incidence rate of ETBF infection. This finding is in agreement with reports from previous studies that indicated poultry or domestic cattle could act as reservoirs for ETBF, with ETBF being transmitted to human hosts through animal dung or poultry feces.^22^ The role of domestic animals in the transmission of enteropathogens to human hosts has been reported in previous studies.^52^

Additional findings from our study included lack of water access, sanitation, and hygiene (WASH) practices, lack of improved floor material in households, lack of improved drinking water, and lack of improved sanitation being associated with greater incidences of ETBF infection. Such findings were also evidenced in several other studies conducted in MAL-ED settings, where similar protective roles of improved WASH practices were found against infection by other enteropathogens.^53^^,^^54^ Moreover, increased maternal education and female gender were found to exert a protective role against ETBF infections, thus agreeing with reports from concurrent studies done in similar settings.^49^^,^^54^ Although enhanced maternal education may correspond to improved WASH practices and thereby reduce ETBF infections, the role of the female gender in reduced ETBF infections warrants further studies into the role of sex chromosomes in conferring protection against these enteropathogens, including ETBF.

In addition, we report a significant protective association of longer duration of breastfeeding with ETBF infections. Longer duration of breastfeeding is protective against the incidence of infections by other enteropathogens in studies carried out in similar settings.^54^ We hypothesize that human milk oligosaccharides, along with maternal antibodies and milk glycans, act in reducing infection by enteropathogens by promoting the growth of probiotic microbes in the gut, thus impeding the growth of pathogenic microorganisms.^55^^,^^56^ Also, increased crowding in households (more than two people living in a single room and mother having more than three living children) was found to be associated with the risk of incidence of ETBF infection, which could be explained by an increased fecal–oral transmission of enteropathogens, accentuated by the lack of access to proper sanitation, as evidenced in such settings.

In our study, we report a positive association between the burden of ETBF infection and EED score at 24 months of age. The relevance of an elevated EED score at 24 months of age with the burden of ETBF infection is yet to be elucidated, and there is no reported indication of enteric inflammation being associated with ETBF infection. However, it is well reported that the toxin secreted by ETBF, known as fragilysin, induces intestinal inflammation that activates β-catenin signaling and induces IL-8 secretion in colonic epithelial cells, leading to neutrophil infiltration in the small intestine and T-cell-mediated hyperstimulation of the gut immune system.^21^^,^^57^^,^^58^ Thus, persistent ETBF infection may lead to sustained secretion of fragilysin from birth over a period of 24 months, which may lead to continual inflammation of the gut epithelium, leading to a gradual change in the architecture of the small intestine.

Our study findings also demonstrate that the burden of ETBF infection was associated negatively with linear growth (LAZ) at 24 months of age. Although other studies conducted in MAL-ED settings reported that linear growth faltering at 24 months of age was associated with sub-clinical infections by certain other pathogens, none of those studies evaluated enteropathogen burden using the incidence rate of the enteropathogen infections at each successive follow-up over the entire study period.^40^ The effect size of the association of infection by other malnutrition-associated co-pathogens (*Campylobacter*,* Shigella*, ETEC, EAEC, tEPEC, and *Giardia*) with the outcome variables of EED score and LAZ at 24 months was greater than that of ETBF infection, as indicated in Table 3. Nevertheless, this is the first report to show a statistically significant association between the burden of ETBF infection and growth faltering at 24 months of age, thus warranting greater clinical vigilance in the management of ETBF infections to reduce the burden of enteropathy and linear growth faltering in childhood.

We can suggest that persistent ETBF infection leading to augmented enteric inflammation, as observed in our study, may have resulted in reduced intestinal barrier function and a decline in the surface area of the villi for absorption of nutrients, which—in conjunction with frequent diarrheal episodes during this period—may possibly have resulted in linear growth faltering at 24 months of age.^59^ Moreover, poor dietary protein quality during this critical period for growth and development, as evidenced in studies in low- to middle-income countries, may have led to a deficiency in circulating amino acids such as tryptophan.^60^^–^^62^ This phenomenon may have resulted in the suppression of intracellular master growth regulation or the mammalian target of rapamycin pathway, ultimately leading to a reduction in the rate of cell growth and proliferation, and linear growth faltering.^60^^,^^63^ However, such dietary data were unavailable for our analysis.

Despite the promising nature of our findings, there are some possible limitations associated with our analysis. First of all, we were unable to establish any conclusive causality between the burden of ETBF infection and EED and linear growth faltering. Thus, a definite biological explanation for our study findings cannot be drawn. In addition, small intestinal endoscopy, the gold standard for diagnosis of EED, was not performed, so we do not have any pertinent data regarding any possible molecular or immunological aberrations in the upper gastrointestinal biopsy specimens of the children in our study. Moreover, were unable to establish a temporal relationship between the ETBF infections and the final study outcomes, which would require structured longitudinal models.

## CONCLUSION

The highest and lowest incidence of ETBF infection per 100 child-months was reported in Tanzania and Peru, respectively. Female gender, longer duration of breastfeeding, and improved WASH practices (such as improved drinking water source, improved sanitation, and improved floor material in households), along with enhanced maternal education and less crowding in the households (more than two people living in a single room and mother having more than three living children) were observed to be protective against incidences of ETBF infection. The burden of ETBF infections was observed to have significant associations with EED and subsequent growth faltering at 24 months of age across all study sites. Our study findings illustrate the clinical significance of ETBF infection, highlighting the long-term adverse effects exerted by asymptomatic gut colonization of ETBF in young children, and in turn may lead to greater monitoring and clinical vigilance in the management of ETBF infections during early childhood to avert the consequent long-term sequelae on a global scale.
